# Professor Silliman

**Published:** 1851-06

**Authors:** 


### PROFESSOR SILLIMAN.

We mentioned, in a former number, that Professor Silliman was traveling in Europe for professional improvement. At our last dates he was in Marseilles. He is expected to return early in October. While abroad he will make purchases for the medical deparement of the University of Louisville.

### CLINICAL LECTURES.

**Dr. D. W. Yandell has commenced a course of Clinical Lectures at the Louisville Marine Hospital, to which we would call the attention of medical students.**

